# Exploring the Promise of Flavonoids to Combat Neuropathic Pain: From Molecular Mechanisms to Therapeutic Implications

**DOI:** 10.3389/fnins.2020.00478

**Published:** 2020-06-09

**Authors:** Md. Sahab Uddin, Abdullah Al Mamun, Md. Ataur Rahman, Md. Tanvir Kabir, Saad Alkahtani, Ibtesam S. Alanazi, Asma Perveen, Ghulam Md Ashraf, May N. Bin-Jumah, Mohamed M. Abdel-Daim

**Affiliations:** ^1^Department of Pharmacy, Southeast University, Dhaka, Bangladesh; ^2^Pharmakon Neuroscience Research Network, Dhaka, Bangladesh; ^3^Center for Neuroscience, Brain Science Institute, Korea Institute of Science and Technology, Seoul, South Korea; ^4^Department of Pharmacy, Brac University, Dhaka, Bangladesh; ^5^Department of Zoology, College of Science, King Saud University, Riyadh, Saudi Arabia; ^6^Department of Biology, Faculty of Sciences, Univesity of Hafr Al Batin, Hafr Al Batin, Saudi Arabia; ^7^Glocal School of Life Sciences, Glocal University, Saharanpur, India; ^8^King Fahd Medical Research Center, King Abdulaziz University, Jeddah, Saudi Arabia; ^9^Department of Medical Laboratory Technology, Faculty of Applied Medical Sciences, King Abdulaziz University, Jeddah, Saudi Arabia; ^10^Department of Biology, College of Science, Princess Nourah bint Abdulrahman University, Riyadh, Saudi Arabia; ^11^Pharmacology Department, Faculty of Veterinary Medicine, Suez Canal University, Ismailia, Egypt

**Keywords:** neuropathic pain, neuronal injury, flavonoids, benzodiazepines, GABA

## Abstract

Neuropathic pain (NP) is the result of irregular processing in the central or peripheral nervous system, which is generally caused by neuronal injury. The management of NP represents a great challenge owing to its heterogeneous profile and the significant undesirable side effects of the frequently prescribed psychoactive agents, including benzodiazepines (BDZ). Currently, several established drugs including antidepressants, anticonvulsants, topical lidocaine, and opioids are used to treat NP, but they exert a wide range of adverse effects. To reduce the burden of adverse effects, we need to investigate alternative therapeutics for the management of NP. Flavonoids are the most common secondary metabolites of plants used in folkloric medicine as tranquilizers, and have been claimed to have a selective affinity to the BDZ binding site. Several studies in animal models have reported that flavonoids can reduce NP. In this paper, we emphasize the potentiality of flavonoids for the management of NP.

## Introduction

Neuropathic pain (NP) is caused by damage or disease affecting the somatosensory nervous system (SSNS) (Colloca et al., 2017; Murnion, 2018). NP may be connected with aberrant sensations, known as dysesthesia, or pain from usually non-painful stimuli, called allodynia. The SSNS plays a pivotal role in the transfer of noxious stimuli to the central nervous system (CNS) under normal circumstances (Myers and Bennett, 2008). Therefore, lesion of the SSNS leads to the innervations of nerve cells stopping and causes pain with or without a sensory hypersensitivity event in the painful region (Jensen and Baron, 2003). Furthermore, an injury in the SSNS could reveal itself as negative sensory symptoms or positive sensory symptoms. The positive sensory symptoms occur because of the regeneration as well as disinhibition of the nerve cells, whereas the negative sensory symptoms occur owing to the partial or complete loss of input to the nervous system (Shehla, 2019; von Hehn et al., 2012). Moreover, positive symptoms can be either spontaneous or stimulus-induced.

Paresthesia (i.e., aberrant sensations of the skin including tingling, chilling, numbness, burning, and pricking), spontaneous or shooting stimulus-independent pain, as well as electric shock-like sensations are involved in spontaneous positive symptoms; whereas, stimulus-induced positive symptoms of neuropathy include allodynic and hyperalgesia pain (Rasmussen et al., 2004; Beran, 2015). On the other hand, hypoesthesia (i.e., decreased sensations to non-painful stimuli), hypoalgesia (i.e., decreased sensations to toxic stimuli), pallhypesthesia (i.e., decreased sensations to vibration), and thermohypoesthesia (i.e., decreased sensations to cold/warm stimuli) are negative symptoms of NP (Jensen and Baron, 2003; Toh et al., 2018).

Many studies have revealed the prospective efficacy of phenytoin, mexiletine, dextromethorphan, tricyclic anti-depressants, gabapentin, tramadol, pregabalin, opioids, and lamotrigine for painful sensory neuropathy (Harden, 1999; Attal, 2001). Conversely, these treatments cause a 30–50% decline in pain and are frequently restricted owing to their noticeable adverse effects, with dominant sedative action. Nowadays, natural products like plant secondary metabolites are widely used to treat several chronic diseases due to their limited adverse effects as well as high efficacy (Uddin et al., 2018b, 2020e; Begum et al., 2019; Samsuzzaman et al., 2019; Thangapandiyan et al., 2019; Basu and Basu, 2020). Flavonoids are a broad group of secondary metabolites, extensively found in many fruits, vegetables, wine, cocoa, and tea (Chun et al., 2007; Egert and Rimbach, 2011). Flavonoids are recognized to have antioxidant, analgesic, and anti-inflammatory properties (Uddin and Upaganlawar, 2019). Moreover, these effects are associated with the suppression of nuclear factor kappa B (NF-κB)-dependent pro-inflammatory cytokines (Borghi et al., 2018), vascular endothelial growth factor, intercellular adhesion molecule 1, signal transducer and activator of transcription 3 (Verri et al., 2012), and activation of antioxidant transcription factor including nuclear factor erythroid 2-related factor 2 (Nrf2) (Borghi et al., 2018).

Numerous flavonoids have been demonstrated to be safe natural alternative treatments against neuropathic pain, oxidative stress, and neuroinflammatory diseases (Azevedo et al., 2013; Quintans et al., 2014; Anusha et al., 2017; Carballo-Villalobos et al., 2018; Ginwala et al., 2019). Hence, flavonoids are considered as multi-target drugs, which elucidate their wide range of actions. Here, we have reviewed the recent studies on the promising effects of flavonoids for the treatment of NP.

## Mechanisms of Neuropathic Pain

**FIGURE 1:**
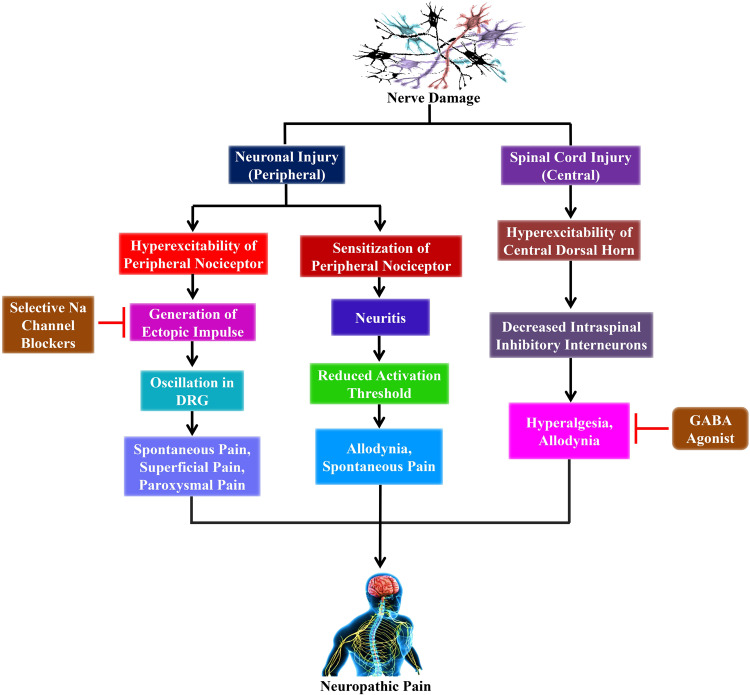
The outlines of the mechanism of neuropathic pain from nerve damage with probable clinical interventions. Nerve damage leads to peripheral nerve injury as well as spinal cord injury (central). The peripheral sensitization and hyperexcitability take place due to the peripheral nerve injury. Furthermore, the hyperexcitability of peripheral nociceptor leads to the generation of ectopic impulses, which plays a crucial role in producing spontaneous pain, superficial pain, and paroxysmal pain that ultimately leads to neuropathic pain. Conversely, selective sodium (Na) channel blockers such as lidocaine and carbamazepine inhibit the generation of ectopic impulses that reduces the sensation of neuropathic pain. On the other hand, the hyperexcitability of the central dorsal horn is caused by spinal cord injury that subsequently decreases intraspinal inhibitory interneurons, which finally leads to neuropathic pain. However, GABA agonists, including baclofen, inhibit the decreased intraspinal inhibitory interneurons that plays an essential role in reducing the sensation of neuropathic pain. DRG, dorsal root ganglion; Na, sodium.

Copious research in animal models have delivered some hint as to the pathophysiological mechanisms that produce NP, which involves both central and peripheral mechanisms (Baron, 2006; Campbell and Meyer, 2006; Gilron et al., 2006) as shown in Figure 1. Furthermore, the peripheral sensitization is performed through unmyelinated C- as well as finely myelinated Aδ-primary afferent neurons, which usually produce the sensation of pain in response to noxious stimuli. Conversely, the peripheral nerve injuries sensitize these neurons that develop a spontaneous activity. Moreover, these injuries result in significant alterations on the molecular and cellular levels activating the nerve cells (Baron, 2006).

Overexpression of messenger ribonucleic acid (mRNA) for voltage-gated sodium channels in the primary afferent neurons is accountable for ectopic spontaneous activity after nerve damage. This event might cause the clustering of these channels, which declines the action potential threshold, leading to hypersensitivity. Therefore, sodium channel blockers, including lidocaine, demonstrate pain relief action in NP through this mechanism (Lai et al., 2003).

Peripheral nerve injury is also responsible for the upregulation of various receptor proteins. These receptors are usually found at the membranes of the primary afferents and are partly expressed during physiological conditions. Vanilloid receptors, including the transient receptor potential cation channel subfamily V member 1 (TrpV1), play a crucial role in the sensing of toxic heat exceeding 43°C (Patapoutian et al., 2003), while transient receptor potential cation channel subfamily M (melastatin) member 8 (TRPM8) has been recognized as cold and menthol-sensitive which increase at temperature ranges from 8 to 28°C. Furthermore, the TRPM8 receptor is expressed in neurons that are small in diameter from the dorsal root ganglia (McKemy et al., 2002). Nerve injuries can cause the upregulation of this channel, contributing to peripheral sensitization of C-nociceptors, which results in cold hyperalgesia (Wasner, 2004).

In addition, acid-sensing ion channels are thought to take part in static mechanical hyperalgesia (Price et al., 2001). In contrast, both α_1_- and α_2_-adrenoceptors situated on the cutaneous afferent fibers play an essential role in hypersensitivity from nerve damage (Baron et al., 1999). Furthermore, adrenergic sensitivity has extensively been expressed in complex regional pain syndromes II, post-traumatic neuralgias, and postherpetic neuralgias; while there is no sensitivity in the primary afferent neurons, which have been claimed in the case of polyneuropathies (Schattschneider et al., 2006). Therefore, sympathetically-induced and temperature-mediated pain can be cured by inhibiting their relevant receptors on nociceptive neurons.

Ectopic activity is mediated by inflammation in both injured and contiguous typical primary afferent nociceptors, which are activated by nerve damage that generates proinflammatory cytokines, particularly tumor necrosis factor-α (TNF-α) (Sommer, 2003). Furthermore, deep proximal, as well as paroxysmal pains are noticeable symptoms in the case of patients who have peripheral neuropathies, including human immunodeficiency virus-neuropathy. Increased concentrations of proinflammatory cytokines and cyclooxygenase-2 (COX-2) have been found in the nerve biopsy specimens of these patients (Lindenlaub and Sommer, 2003).

CNS forms precise anatomical connections with the thalamus, brain stem, cortex, and spinal cord. Furthermore, these relations can connect the sensations that are produced in the high threshold primary afferents with the cortical areas of the CNS, which subsequently processes it into final painful sensations (Woolf, 2011). The constant hyperactivity is produced by damaged nerves that are considered to be a causative factor for central sensitization, as well as triggering activity-dependent synaptic flexibility occurring inside the cortex. Moreover, various factors are involved in central sensitization such as excitatory amino acid, changes in ion channel kinetics, different synaptic modulators, pre- and post-synaptic activation of kinases, and increased bulk of ionotropic receptors.

Most of the patients who have peripheral as well as central neuropathy demonstrate dominant synaptic facilitation leading to hypersensitivity and allodynia (Campbell and Meyer, 2006). Additionally, peripheral nerve damage results in pre-synaptic changes such as alterations in the synthesis of neuromodulators, neurotransmitters, and modifications in the calcium channels density (Hendrich et al., 2008). In contrast, post-synaptic changes take place due to the increased density of receptors on account of increased synthesis of ion channels and scaffold proteins and the phosphorylation of N-methyl-D-aspartate (NMDA) subunits (Cheng et al., 2008). These alterations also lead to aberrant expression of the mitogen-activated protein kinase system and Nav 1.3 (Hains et al., 2004; Ji and Woolf, 2001). Furthermore, pathologically sensitized C-fibers sensitize neuropeptide substance P as well as a spinal dorsal horn (SDH) through the release of glutamate, which cannot be neglected. Subsequently, glutamate demonstrates an excitatory action by acting upon the postsynaptic NMDA receptor contributing to central sensitization (Ultenius et al., 2006). It has been observed that the involvement of loss of function of tonic γ-amino butyric acid-A (GABA_A_)-conciliated inhibition and enhanced excitatory neurotransmitters are caused by an induction of central sensitization, leading to peripheral hypersensitivity, specifically hyperalgesia and allodynia (Knabl et al., 2008). When this sensitivity is developed, the generally harmless tactile stimuli can trigger Aβ as well as Aδ low threshold mechanoreceptors (Tal and Bennett, 1994).

## GABA and Neuropathic Pain

The most abundant inhibitory neurotransmitter in the brain is GABA (Uddin et al., 2018a). GABA regulates diverse physiological functions such as anxiety, sleep, reward, and memory formation (Zeilhofer et al., 2009; Spiering, 2018; Uddin and Amran, 2019). GABA also regulates the excitatory action of neuronal cells of the CNS, assisting and maintaining the neural circuit’s homeostasis. Previously, it has been described that the role of inhibitory neurons, especially in SDH, act and monitor transmission of pain via the periphery to greater intensities of the brain (Melzack and Wall, 1965). After this, GABA was established to be the primary inhibitory neurotransmitters in the brain’s SDH (Yaksh, 1989).

GABA, releasing from presynaptic neurons, acts postsynaptically with several receptors; G protein-coupled channels, GABA_A,_ GABA_B,_ as well as GABA_C,_ are ligand-gated ion channels (Gavande et al., 2011). Generally, ionotropic GABA_A_ receptors are comprised of 5 heteropentameric subunits that form transmembrane protein complexes (Uddin and Rashid, 2020). Meanwhile, the α_1_β_2_γ_2_ subunit is thought to be the most dominant one in the human brain (Wafford, 2005). GABA initiation stimulates the membrane penetrability to chloride and carbonate ions that produce a net inner flow of anions as well as resulting in hyperpolarization. Therefore, this hyperpolarizing post-synaptic reaction is known as inhibitory post-synaptic potential (Semyanov et al., 2003).

Physiologically, GABA-liberating interneurons impose a robust inhibitory regulation through dorsal horn neuronal cells. Besides, damage of these neurons might additionally stimulate the dominant sensitization of the models of peripheral partial nerve injury. In rodents, injury caused the reduction of GABA release from the spine, with reduced GABA-producing glutamic acid decarboxylase (Moore et al., 2002). However, in diseased conditions, an improved excitation state arises that is recognized as an enormous GABAergic neuronal loss or deterioration of interneurons. Therefore, an imbalance of this condition could culminate into several neurological as well as psychiatric diseases such as Alzheimer’s disease, Parkinson’s disease, schizophrenia, epilepsy, NP, and the collective role of inhibitory and excitatory neurons show a dynamic role in regulating many brain activities (Tyson and Anderson, 2014).

It has been found that peripheral and central sensitization causes nerve injury and NP. GABAergic interneuronal loss is considered to be the main contributor to persistent pain states (Bráz et al., 2012). In the spinal cord, pharmacological inhibition of GABAergic neurotransmission causes hyperalgesia and allodynia (Gwak et al., 2006; Jergova et al., 2012). Likewise, GABA_A_ receptor blockage could prompt a behavioral reaction, which was revealed by electrophysiological studies (Hwang and Yaksh, 1997). Furthermore, the GABAergic system impaired chronic NP in animals (Zeilhofer, 2008). As a result, spinal inhibitory neurotransmission may be appreciated as a pharmacological NP treatment.

Additionally, the crucial function of GABA in opioid-mediated antinociception has long been recognized (Ossipov et al., 2010). Also, agonists of GABA_A_ receptor-mediated antinociceptive activity have been recognized to stimulate or inhibit additional neurotransmitters (McCarson and Enna, 2014). As a consequence, the agonists of the GABA receptor might play a dynamic role in considering chronic and acute pain (McCarson and Enna, 2014). Incidentally, isoguvacine and muscimol, agonists of GABA_A_ receptors, are described to oppose nerve injury-stimulated tactile allodynia (Hwang and Yaksh, 1997). These receptors are strictly linked to huge diameter afferents involved in innocuous sensation (Price et al., 1984; Sivilotti and Woolf, 1994; Reeve et al., 1998; Ataka et al., 2000; Riley et al., 2001; Turner, 2003). Pharmacological as well as behavioral examinations have stated that a single or continuous intrathecal GABA response to spinal cord or GABA liberating cells reduce NP (Eaton et al., 1999a, b; Stubley et al., 2001; Malan et al., 2002). In addition, spinal GABA_A_ receptors inhibition shows annoying peripheral nerve injury connected to hyperalgesia (Yamamoto and Yaksh, 1993).

In contrast, intrathecal administration of benzodiazepines (BDZs) and allosteric positive modulators of GABA_A_ receptors have been extensively used in sleep complaints, convulsions, anxiety, and analgesic activity (Tucker et al., 2004). Even though it has analgesic properties, its usage in pain relief is limited due to sedation. Therefore, study is urgently needed in to GABAergic modulators which might play a prominent role in the attenuation of NP.

## Flavonoids

**FIGURE 2:**
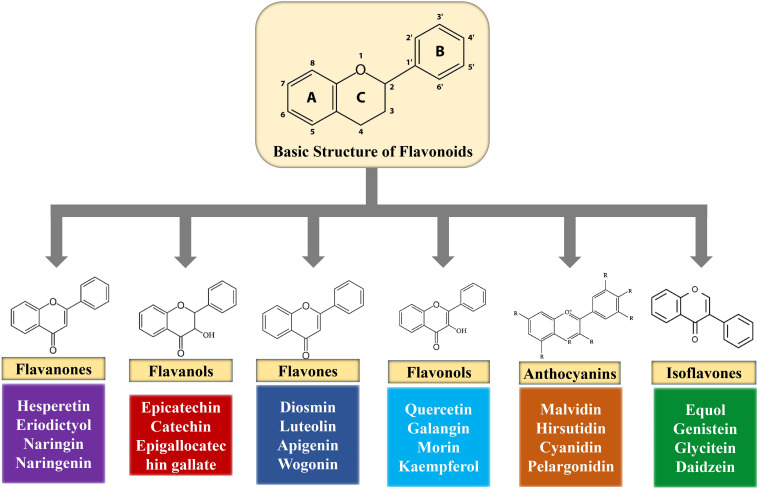
Basic structure of flavonoids and their sub-classes.

Flavonoids are polyphenolic compounds found in fruits, flowers, barks, grains, vegetables, roots, tea, stems, and so on (Uddin et al., 2020a). Chemically, flavonoids are 15-carbon skeletons comprising of two benzene rings (A and B) linked via a heterocyclic pyrane ring (C) (Kumar and Pandey, 2013) as shown in Figure 2. Flavonoids can be divided into diverse subgroups according to the carbon of the C ring whereon the B ring is connected as well as the oxidation and degrees of unsaturation of the C ring (Panche et al., 2016).

In 1930, a novel constituent derived from an orange was believed to be a vitamin and called vitamin P. It was subsequently proved to be a flavonoid, rutin, that played an essential role in the isolation as well as the study of the mechanisms of several individual flavonoids. In fact, several traditional medicines are mainly flavonoids. In past centuries, *Tanacetum parthenium* has been used as a prophylactic drug in the treatment of migraine, while *Matricaria recutita*, chamomile flowers, has been used as a tranquilizer for many decades, with both comprising of the active constituent apigenin (Jäger et al., 2009). Moreover, linden flowers, *Tilia sp. Tiliaceae*, have been used as sedative agents, and *Calluna vulgaris* might serve as a nerve-calming medicine, which has active components of kaempferol and quercetin (Aguirre-Hernández et al., 2010). Apart from the separation of natural flavonoids, several synthetic and semi-synthetic products have been synthesized and separated for their therapeutic potential (Cushnie and Lamb, 2005). Up to now, 6000 diverse flavonoids have been isolated. Flavonoid compounds show different biological effects, such as neuroprotective (Cho et al., 2013; Uddin et al., 2016; Uddin and Kabir, 2019; Zaplatic et al., 2019), antifungal (Ammar et al., 2013), antimicrobial (Cushnie and Lamb, 2005; Górniak et al., 2019), anticancer (Liu et al., 2010; Abotaleb et al., 2019), anti-inflammatory (Wang et al., 2010; Begum et al., 2019), anxiolytic (Ognibene et al., 2008), antioxidant (Heim et al., 2002; Uddin et al., 2017), antiviral (Orhan et al., 2010; Dai et al., 2019), cardioprotective (Yu et al., 2005; Mahmoud et al., 2019), and antinociceptive activities (Wang et al., 2014; Hossain et al., 2017a, b).

## Role of Flavonoid on Ionotropic GABA_A_ Receptors

Flavonoids are widely targeted for their peripheral events; though, their selective affinity for GABA_A_ receptors has extensively been demonstrated in studies using bovine and rat brain membrane binding analyses (Hong and Hopfinger, 2003). Numerous behavioral tests have also widely been performed, which confirm the sedative effects of flavonoids in an animal model of anxiety that was devoid of the additional side effects of BDZs (Griebel et al., 1999). Remarkably, negative, positive, and neutral allosteric modulatory flavonoid actions of an extensive variety of ionotropic GABA receptors have been focused on and intensely supported through enormous evidence. In the 1990s, flavonoids had been well-defined as a novel family of BDZ receptor ligands (Medina et al., 1997; Marder and Paladini, 2002). Typically, they were believed to be acting upon BDZ receptors, as well as many synthetic flavonoids having a remarkable affinity for BDZ binding site (Yao et al., 2007), until they were claimed to be insensitive to the BDZ receptor antagonist, flumazenil, therefore focusing a distinctive site of action (Hanrahan et al., 2011).

It has been found that the replacement at 6- or 3′-positions of flavones with an electronegative functional group improved the affinity toward the receptors of BDZ (Paladini et al., 1999). Moreover, GABA ratios were measured by the impact of ligand binding on the GABA binding site. These ratios displayed that flavones showed substantial biological actions at BDZ receptors (Hanrahan et al., 2011). 6-Bromo-3′-nitroflavone, 6-chloro-3′-nitroflavone, and 6-bromoflavone with a GABA ratio of 1.38, 2.0, and 1.6–2.0 were demonstrated as a partial agonist, an antagonist, and full agonist at these receptors respectively (Marder et al., 1996; Wolfman et al., 1998; Viola et al., 2000).

GABA_A_ receptors were enhanced by the flux of chloride ion that deliver a robust inhibitory effect via positive ionotropic modulators. As a result, these modulators are the strongest candidates for the management of CNS-associated diseases such as generalized anxiety, seizure disorders, sleep disturbances, panic disorders, muscle spasms, and NP (Rudolph and Möhler, 2006). Furthermore, flavonoids might act upon a new binding site, excluding the classical BDZ binding site, which plays a pivotal role in searching for novel therapeutic candidates with limited adverse effects (Rudolph and Möhler, 2006). Incidentally, 6-methoxyflavonone has been described to act as a positive allosteric modulator at α1β2γ2L and α2β2γ2L subunits of GABA_A_ receptors (Hall et al., 2014).

The substitution at 6-position on flavones is linked to its role in recombinant GABA_A_ receptors. 6-Hydroxyflavone showed a remarkable effect at the flumazenil-sensitive BDZ site (Ren et al., 2010). Furthermore, 6-methoxyflavone and 6-methoxyflavanone have been claimed to display anti-allodynic effects in cisplatin- and streptozotocin-stimulated NP models (Akbar et al., 2016; Shahid et al., 2017). Therefore, these defensive properties against NP have been recognized to cause allosteric positive modulatory effects on opioid and GABA_A_ receptors (Akbar et al., 2016).

Additionally, myrcitin and baicalin exerted antiallodynic effects in sciatic nerve ligation models (Cherng et al., 2014; Meotti et al., 2006). Besides, quercetin and rutin have widely been claimed to suppress oxaliplatin-mediated chronic peripheral neuropathic pain (Azevedo et al., 2013). Meanwhile, the antiallodynic potential of streptozotocin-induced painful diabetic neuropathy has been reported by naringin (Kandhare et al., 2012).

## Role of Flavonoids in Different Neuropathic Pain Models

### Effect of Flavonoids on Diabetic Neuropathy

NP is arduous to treat properly and is related to the remarkable impairment of health conditions as well as economic problems (O’Connor, 2009; Langley et al., 2013). Diabetic neuropathy is one of the most common causes of neuropathy and affects about 382 million people in the world (Boulton et al., 1998). Furthermore, genistein (Valsecchi et al., 2011), luteolin (Li et al., 2015), catechin (Addepalli and Suryavanshi, 2018), rutin (Tian et al., 2016), and pelargonidin (Mirshekar et al., 2010) have been revealed to decrease the levels of malondialdehyde (MDA) in animal models of diabetes. Moreover, MDA serves as a key biomarker for lipid damage as well as oxidative stress that can be caused by free radicals. In diabetic patients, the increased level of MDA has widely been observed in the serum as well as other tissues, which significantly affects the peripheral nerves (Feldman et al., 1994; Perkins et al., 2001). Some flavonoids, such as genistein (Valsecchi et al., 2011), naringenin (Al-Rejaie et al., 2015), luteolin (Li et al., 2015), hesperidin, catechin (Addepalli and Suryavanshi, 2018), kaempferol (Kishore et al., 2018), fisetin (Zhao et al., 2015), rutin (Tian et al., 2016), and morin (Bachewal et al., 2018) have been shown to reduce the ROS level by increasing the level of diverse antioxidative enzymes including glutathione peroxidase, reduced glutathione peroxidase, superoxide dismutase, glutathione reductase, and catalase in various tissues such as the liver, sciatic nerve, and brain of diabetic animals (Table 1). In the diabetic animal model, rutin, luteolin, and morin have been demonstrated to raise the expression of Nrf2 as well as its downstream effector’s heme oxygenase-1 (HO-1) in nerve tissues. Numerous studies have found that Nrf-2/HO-1 could fight against oxidative stress-mediated neuroinflammation and nerve damage in diabetic animal models (Cardozo et al., 2013; Agca et al., 2014; Kumar and Mittal, 2017). Moreover, kaempferol decreased advanced glycation end products and epigallocatechin gallate (EGCG) causes a reduction of 8-hydroxy-2-deoxyguanosine, which is considered as the major form of free radical-mediated oxidative stress in the nucleus and mitochondria (Valavanidis et al., 2009). It has also been found that in the diabetic animal model, genistein and naringenin raised nerve growth factor (NGF) in sciatic nerves (Basu and Basu, 2020). Therefore, NGF servesas the survival and life maintenance of the neurons.

**TABLE 1:** Promising studies of flavonoids for the management of neuropathic pain.

Flavonoids	Species/studied materials	Experimental model	Dose	Route of administration	Effects	References
Genistein	C57BL/6J male mice	Chronic constriction sciatic nerve injury	1, 3, 7.5, 15, and 30 mg/kg	Subcutaneous injection	Ameliorate painful neuropathy by decreasing the mRNA expressions of IL-1β and IL-6 in sciatic nerve as well as protein expression of IL-1β in dorsal root ganglion and spinal cord	Valsecchi et al., 2008
	Male C57BL/6J mice	Streptozotocin-induced diabetic	3 and 6 mg/kg	Subcutaneous injection	Ameliorates diabetic peripheral neuropathy by inhibiting proinflammatory cytokine and the overproduction of reactive oxygen species, as well as restored the NGF content in diabetic sciatic nerve	Valsecchi et al., 2011
	Male Sprague-Dawley rats	High-fat diet	4 and 8 mg/kg/day	Intragastrical	Decreases the levels of TNF-α and IL-6 in serum that produce anti-inflammatory actions	Ji et al., 2011
Quercetin	Male albino mice	Streptozotocin-induced diabetic	50 and 100 mg/kg	Oral	Antinociceptive activity via the modulation of opioidergic mechanism that attenuates diabetic neuropathic pain	Anjaneyulu and Chopra, 2003
	Male Sprague−Dawley rats	Streptozotocin-induced diabetic	10 mg/kg	Oral	Effective in diabetic neuropathy	Anjaneyulu and Chopra, 2004
	Male Sprague-Dawley rats and mice	Paclitaxel-induced neuropathic pain	20 and 60 mg/kg – *in vivo* and 3, 10, 30 μM/L – *in vitro*	Intraperitoneal injection	Ameliorates neuropathic pain by decreasing the levels of protein kinase C (PKC)ε and TrpV1 in the spinal cord dorsal horns and dorsal root ganglions	Gao et al., 2016
Quercetin and rutin	Male Swiss mice	Oxaliplatin-induced peripheral neuropathy	Rutin and quercetin (25, 50, and 100 mg/kg)	Intraperitoneal injection	Ameliorates peripheral neuropathy	Azevedo et al., 2013
Myricitrin	Adult Swiss mice	Partial Sciatic Nerve Ligation	30 mg/kg	Intraperitoneal injection	Antinociceptive activity via the inhibition of PKC and nitric oxide cell signaling	Meotti et al., 2006
	Adult male Wistar rats	Spinal nerve ligation	0.1, 1 and 10 mg/kg	Intraperitoneal injection	Reduces neuropathic pain that might be related to its PKC-induced decrease of voltage-gated calcium channel currents in dorsal root ganglia neurons	Hagenacker et al., 2010
Epigallocatechin gallate	Adult male Wistar rats	Alcoholic neuropathy	25, 50, 100 mg/kg	Oral	Reduces neuropathic pain through the modulation of oxido-inflammatory pathway	Tiwari et al., 2011
	Male Sprague-Dawley rats	Chronic constriction injury	1 mg/kg	Intrathecal injection	Ameliorates neuropathic pain through the suppression of TLR4 signal pathway that reduces the expressions of NF-κB, IL-1β and TNF-α	Kuang et al., 2012
	Male Wistar rats	Streptozotocin-induced diabetic	2 g/L	Oral gavage	Ameliorates diabetic neuropathy by preventing oxidative stress	Raposo et al., 2015
Puerarin	Male Sprague-Dawley rats	Chronic constriction injury	100 mg/kg/day	Intraperitoneal injection	Reduces neuropathic pain through the P2X3 receptors in dorsal root ganglion neurons	Xu et al., 2012
	Male Sprague-Dawley rats	Chronic constriction injury	4, 20, and 100 nM	Intrathecal injection	Reduces neuropathic pain by the inhibition of spinal NF-κB activation and the upregulation of cytokines	Liu et al., 2014
2″− O− rhamnosylswertisin	Female Swiss and C57/BL6 mice	Partial Sciatic Nerve Ligation	125, 250 or 500 mg/kg	Oral	Antinociceptive activity by reducing the neutrophil migration and IL-1β levels	Quintão et al., 2012
Naringin	Adult male Wistar rats	Streptozotocin-induced diabetic	20, 40, and 80 mg/kg	Oral	Reduces neuropathic pain by down−regulation of cytokine including TNF-α	Liu et al., 2017
	Wistar rats.	Cisplatin-Induced Cognitive Dysfunction	25, 50, and 100 mg/kg	Oral gavage	Ameliorates neuropathic pain through the involvement of oxidative-stress-mediated inflammatory signaling	Chtourou et al., 2015
	Male Wistar rats	Streptozotocin-induced diabetic	40 and 80 mg/kg	Intraperitoneal injection	Ameliorates diabetic neuropathy by downregulation of free radical, cytokine mediator including TNF-α	Kandhare et al., 2012
	Male Wister albino rats	Streptozotocin-induced diabetic	25 and 50 mg/kg/day	Intraperitoneal injection	Ameliorates diabetic neuropathy through its antioxidant and anti-inflammatory properties	Al-Rejaie et al., 2015
Icariin	Male Sprague- Dawley rats	Paclitaxel-induced neuroinflammation and peripheral neuropathy	25, 50, and 100 mg/kg	Intrathecal injection	Reduces neuropathic pain by the level of TNF-α, IL-1β, and IL-6, astrocytes, NF-κB (p65) phosphorylation in spinal cord	Gui et al., 2018
6-Methoxyflavone	Male Sprague- Dawley rats	Chemotherapy-induced peripheral neuropathy	25, 50 and 75 mg/kg	Intraperitoneal injection	Reduces neuropathic pain	Shahid et al., 2017
	Female Sprague- Dawley rats and BALB/c mice	Streptozotocin-induced diabetic	10 and 30 mg/kg	Intraperitoneal injection	Attenuates neuropathic pain through interactions with the GABAergic and opioidergic systems	Akbar et al., 2016
Catechin	Male Sprague- Dawley rats	Streptozotocin-induced diabetic	25 mg/kg and 50 mg/kg	Intraperitoneal injection	Attenuation of diabetic autonomic neuropathy through the improvement in antioxidant enzymes in vagus nerves	Addepalli and Suryavanshi, 2018
Morin	Male Sprague- Dawley rats	Streptozotocin-induced diabetic	50 and 100 mg/kg – *in vivo*, 10 and 20 μM – *in vitro*	Oral gavage	Reduces diabetic neuropathy by inhibiting NF−κB−mediated neuroinflammation and increasing Nrf2−mediated antioxidant defenses in high glucose−induced N2A cells	Bachewal et al., 2018
	Male Sprague- Dawley rats	Chronic constriction injury	15 and 30 mg/kg	Oral gavage	Ameliorates neuropathic pain by decreasing the inflammatory markers (PARP, iNOS, COX-2, NF-κB and phospho-NF-κB, TNF-α, and IL-6) in the spinal cord	Komirishetty et al., 2016
Kaempferol	Male Wistar rats	Streptozotocin-induced diabetic	5 and 10 mg/kg		Reduces diabetic neuropathy by attenuating oxidative stress-mediated release of pro-inflammatory cytokines	Kishore et al., 2018
Rutin	Male Sprague- Dawley rats	Streptozotocin-induced diabetic	5, 25, and 50 mg/kg	Intraperitoneal injection	Ameliorates diabetic neuropathy through the up-regulation of the expression of Nrf2	Tian et al., 2016
Baicalin	Male Sprague- Dawley rats	Streptozotocin-induced diabetic	10, 20, and 40 μg/kg	Intraperitoneal injection	Analgesic activity in diabetic neuropathic pain through transient receptor potential vanilloid 1	Li et al., 2018
	C57Bl6/J mice	Streptozotocin-induced diabetic	30 mg/kg	Intraperitoneal injection	Reduces diabetic peripheral neuropathy via the suppression of oxidative-nitrosative stress as well as p38MAPK activation	Stavniichuk et al., 2011
Luteolin	Male Sprague- Dawley rats	Streptozotocin-induced diabetic	50, 100, and 200 mg/kg	Intraperitoneal injection	Ameliorates diabetic neuropathy through the up-regulation of the expression of Nrf2	Li et al., 2015
	Male Sprague- Dawley rats	Chronic constriction injury	0.1–1.5 mg	Intrathecal or intracerebroventricular injection	Reduces mechanical and cold hyperalgesia by activating GABA_A_ receptors in a flumazenil-insensitive manner as well as μ-opioid receptors in the spinal cord	Hara et al., 2014
Fisetin	Male C57BL/6J mice	Chronic constriction injury	10 mg/kg	Oral gavage	Ameliorates chronic neuropathic pain	Zhao et al., 2015
	Male C57BL/6J mice	Chronic constriction injury	5, 15 and 45 mg/kg	Oral gavage	Exerts antinociceptive activity through the serotonergic system (coupled with 5-HT7)	Zhao et al., 2015
Diosmin	Male Swiss mice	Chronic constriction injury	1, 10 mg/kg	Intraperitoneal injection	Ameliorates neuropathic pain by activating the NO/cGMP/PKG/KATP channel signaling	Bertozzi et al., 2017
Hesperidin	Sprague Dawley rats	Streptozotocin-induced diabetic	25, 50 and 100 mg/kg	Oral gavage	Reduces diabetic neuropathy by down-regulating the production of free radical, release of cytokines (TNF-α and IL-1β) and elevation in membrane bound enzyme	Visnagri et al., 2014
Diosmin and hesperidin	Male Wistar rats	Chronic constriction injury	Hesperidin (10, 100, 316.2, 562.3, 1000 mg/kg); Diosmin (10, 100 mg/kg)	Intraperitoneal injection	Ameliorates neuropathic pain by the modulation of D2 dopamine, and opioids receptors	Carballo-Villalobos et al., 2016
Pelargonidin	Male Albino Wistar rats	Streptozotocin-induced diabetic	10 mg/kg	Oral gavage	Ameliorates diabetic neuropathic hyperalgesia via attenuation of oxidative stress	Mirshekar et al., 2010
Isoorientin	Male pathogen-free Institute of Cancer Research (ICR) mice	Chronic constriction injury	7.5, 15, and 30 mg/kg	Intragastrical	Ameliorates neuropathic pain by decreasing the expression of IL-6, IL-1β, and TNF-α levels	Zhang et al., 2019
Grape seed proanthocyanidins	Wistar rats	Chronic constriction injury	100 and 200 mg/kg	Oral gavage	Anti-nociceptive and anti-inflammatory effect by inhibiting the inflammatory pathways	Kaur et al., 2016

**FIGURE 3:**
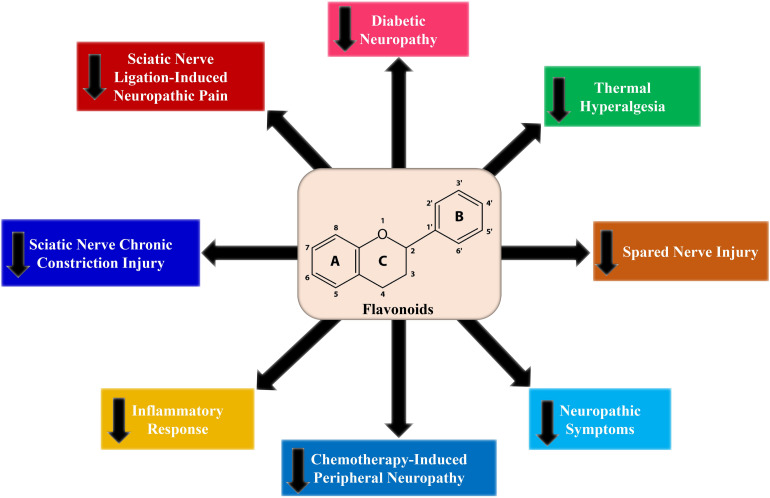
Effects of flavonoids on different neuropathic pain models.

Diabetic neuropathy in animal models has widely been marked by evaluating behavioral signs, such as chemical, mechanical, thermal hyperalgesia, and tactile allodynia (Pittenger et al., 2005). It has also been observed that flavonoids considerably downregulated thermal, mechanical, chemical hyperalgesia, and tactile allodynia in diabetic animal models (Figure 3). A number of flavonoids including fisetin (Zhao et al., 2015), baicalin (Li et al., 2018), naringenin (Al-Rejaie et al., 2015), pelargonidin (Mirshekar et al., 2010), rutin (Tian et al., 2016), naringin (Kandhare et al., 2012), hesperidin (Visnagri et al., 2014), and luteolin (Li et al., 2015) reduced diabetes-mediated thermal hyperalgesia, although kaempferol (Kishore et al., 2018), EGCG (Raposo et al., 2015), rutin (Tian et al., 2016), naringenin (Al-Rejaie et al., 2015), luteolin (Li et al., 2015), morin (Bachewal et al., 2018), and naringin (Kandhare et al., 2012) attenuated mechanical hyperalgesia (Figure 3). Furthermore, fisetin (Zhao et al., 2015), baicalein (Li et al., 2018), hesperidin (Visnagri et al., 2014), morin (Bachewal et al., 2018), and puerarin (Liu et al., 2014) ameliorated mechanical allodynia, while naringin (Kandhare et al., 2012) and genistein (Valsecchi et al., 2011) decreased mechano-tactile allodynia, and rutin (Tian et al., 2016) and luteolin (Li et al., 2015) improved cold allodynia. Numerous investigations have demonstrated that short term diabetes mediated mechanical, chemical, and thermal hyperalgesia (Dyck et al., 2000; Freshwater and Calcutt, 2005), however chronic diabetes induces mechanical and thermal hypoalgesia (Calcutt et al., 2004). Besides, baicalein attenuated thermal hypoalgesia (Stavniichuk et al., 2011).

### Effect of Flavonoids on Chemotherapy-Induced Peripheral Neuropathy

The use of diverse chemotherapeutic agents and other anticancer drugs leads to the impairment of the peripheral nerves. Chemotherapy-induced peripheral neuropathy (CIPN) is another form of neuropathy caused by anticancer drugs (Hershman et al., 2014). Platinum compounds are extensively used in the management of several solid tumors. Oxaliplatin, a third-generation platinum agent, plays a pivotal role in diminishing antitumoral resistance with noticeable cytotoxicity (Argyriou et al., 2008; Stein and Arnold, 2012). It has been observed that (Azevedo et al., 2013) rutin and quercetin suppressed oxaliplatin-mediated mechanical as well as cold nociceptive thresholds. In a study by Schwingel et al. (2014) it was demonstrated that rutin and nanoemulsion of quercetin ameliorated oxaliplatin-mediated mechanical allodynia. According to the study by Shahid et al. (2017) 6-methoxyflavone showed antinociceptive activity in a rat model of CIPN (Table 1). Hence, 6-methoxyflavone considerably reduced cisplatin-mediated mechanical allodynia by raising the paw withdrawal threshold as well as thermal hypoalgesia (Figure 3) by improving the paw thermal threshold.

### Effect of Flavonoids on Sciatic Nerve Chronic Constriction Injury

Chronic constriction injury (CCI) is considered to be the most extensively studied model for chronic neuropathic pain. There are various symptoms of CCI-induced pain including hyperalgesia, allodynia, paraesthesia, dysesthesia, and spontaneous pain (Austin et al., 2012). Flavonoids including hesperidin (Carballo-Villalobos et al., 2016), diosmin (Bertozzi et al., 2017), and grape seed proanthocyanidins (Kaur et al., 2016) decreased both mechanical and thermal hyperalgesia (Figure 3). In contrast, other flavonoids including genistein (Valsecchi et al., 2008), EGCG (Kuang et al., 2012), EGCG-derived compounds (Xifró et al., 2015), morin (Komirishetty et al., 2016), and isoorientin (Zhang et al., 2019) decreased only thermal hyperalgesia (Table 1). As compared to morphine and gabapentin, quercetin decreased the mechanical and thermal hypersensitivities to a greater extent (Çivi et al., 2016). When quercetin was administered in a pre-injury condition, it exerted long term actions on mechanical hypersensitivity, which further suggests the antinociceptive properties of quercetin in the CCI model (Çivi et al., 2016). Additionally, flavonoids including genistein (Valsecchi et al., 2008), EGCG (Kuang et al., 2012), puerarin (Liu et al., 2014), morin (Komirishetty et al., 2016), and isoorientin (Zhang et al., 2019) decreased CCI-mediated mechanical allodynia. On the other hand, cold allodynia was reduced by morin (Komirishetty et al., 2016) and isoorientin (Zhang et al., 2019). Although mechanical and cold hyperalgesia was reduced by luteolin, it did not affect thermal hyperalgesia (Hara et al., 2014). However, thermal hyperalgesia was decreased by fisetin, but did not affect the nociceptive sensitivity to mechanical stimuli (Zhao et al., 2015).

**FIGURE 4:**
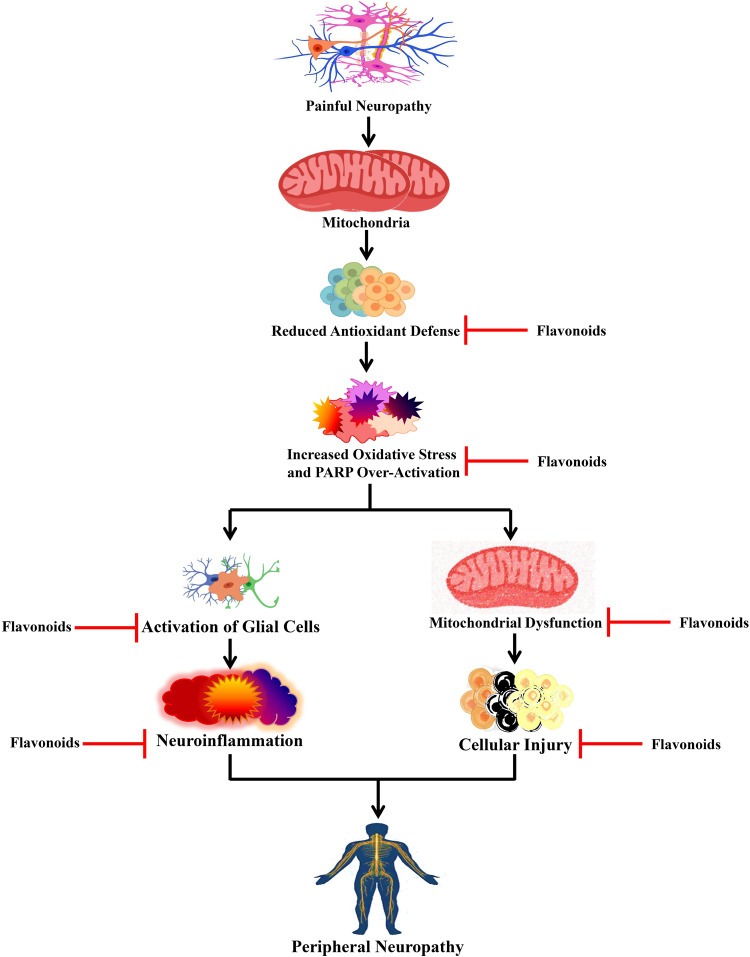
Effects of flavonoids on peripheral neuropathy. Flavonoids act on different peripheral neuropathic pain conditions by blocking oxidative stress, activation of glial cells, and mitochondrial dysfunction. PARP, poly-ADP ribose polymerase.

An elevated level of nitro oxidative stress can cause DNA damage, which can cause the activation of poly-ADP ribose polymerase (PARP) (Figure 4), which can further lead to PARP-induced DNA repair by transferring ADP-ribose units to the nuclear proteins. Nevertheless, activation of PARP can cause NF-κB activation, which can subsequently activate various inflammatory markers including interleukin (IL)-6, TNF-α, inducible nitric oxide synthase (iNOS), and cyclooxygenase-2 (COX-2) (Obrosova et al., 2004; Sommer and Kress, 2004) that lead to neuroinflammation (Figure 4). Studies involving the CCI-induced neuropathic pain model revealed that flavonoids exert effects on various pro-inflammatory biomarkers (Bertozzi et al., 2017; Figure 3). A single administration of diosmin decreased the levels of mRNA expressions of IL-33/ST2 and IL-1β, while chronic administration decreased the mRNA expression level of TNF-α along with ST2, IL-33, and IL-1β. Furthermore, a single administration also decreased the expression levels of oligodendrocytes and microglia, whereas chronic treatment decreased astrocytes together with oligodendrocytes and microglia (Bertozzi et al., 2017). Puerarin decreased the elevated immunoreactivity of glial fibrillary acidic protein and ionized calcium-binding adaptor protein-1, which are astroglia and microglial activation markers, successively (Liu et al., 2014). In the CCI-induced neuropathic pain model, morin decreased various inflammatory biomarkers including IL-6, TNF-α, phospho-NF-κB, NF-κB, COX-2, iNOS, and PARP (Komirishetty et al., 2016). Deoxyribonucleic acid (DNA) damage was found to be increased due to the CCI-induced nerve injury, which resulted in PARP overactivation (Jagtap and Szabo, 2005). It was found that overactivation of PARP caused bioenergetic failure because overactivity of PARP requires a high amount of nicotinamide adenine dinucleotide (NAD) during DNA repair, and finally, NAD synthesis also requires adenosine triphosphate (ATP), which can eventually lead to the disruption of biochemical processes that are dependent on ATP (Hyo and Snyder, 1999).

Treatment with morin caused marked restoration of CCI-mediated reduction in the ATP levels and also restored the neuronal cells from the bioenergetic crisis (Komirishetty et al., 2016). In a study, Kuang et al. (2012) revealed that treatment with EGCG reduced the mRNA and protein expressions of the toll-like receptor (TLR4) and its endogenous ligand HMGB1. It is known that TLR4 is a pattern recognition receptor and plays roles in the immune system and inflammatory diseases. When endogenous ligands bind with TLR4, it gets activated and stimulates the generation of pro-inflammatory cytokines by causing NF-κB activation (Janeway and Medzhitov, 2002; Akira et al., 2006). Furthermore, EGCG elevated the level of IL-10, reduced the downstream pro-inflammatory cytokines (i.e., TNF-α and IL-1β) of the TLR4 signaling pathway, and reduced the expression of NF-κB in the lumbar SDH of CCI rats (Kuang et al., 2012). In the dorsal horn of the spinal cord, an EGCG-derived compound decreased the levels of mRNA and protein expressions of IL-6, NF-κB, IL-1β, and TNF-α (Xifró et al., 2015). Administration of isoorientin and puerarin also decreased the level of CCI-induced pro-inflammatory cytokines including IL-6, IL-1β, and TNF-α (Liu et al., 2014; Zhang et al., 2019). Interestingly, genistein reduced the level of IL-1β expression in the spinal cord and dorsal root ganglion, while genistein also decreased mRNA expressions of both IL-6 and IL-1β in the sciatic nerve (Valsecchi et al., 2008).

### Effect of Flavonoids on Other Neuropathic Pain Signaling Pathways

Flavonoids show anti-inflammatory as well as antioxidant effects due to their action on GABA_A_ receptors (Hanrahan et al., 2011). Maximum metabolic disorders are the result of oxidative stress. Along with exogenous factors, regular metabolism of oxygen inside the tissues and cells produce reactive oxygen species (ROS) and free radicals that steadily endanger them (Forrester et al., 2018; Uddin et al., 2019). Flavonoids are well-recognized for their antioxidant properties and are also confirmed to show beneficial effects in several chronic diseases, including neurodegenerative disease, diabetes, atherosclerosis, and cancer (de Teles et al., 2018; Kozłowska and Szostak-Węgierek, 2019; Uddin and Kabir, 2019; Uddin et al., 2020c, d). Moreover, certain flavonoids play a crucial role in the iron chelation thus stopping the development of free radicals (Nelson et al., 1992; Ferrali et al., 1997). Rutin and epicatechin are shown to have the capability to be oxidized themselves through free radicals, producing a less reactive and stable species (Hanasaki et al., 1994). Correspondingly, quercetin, a plant pigment flavonoid, prevents nitric oxide (NO)-mediated cell injury. A combination of NO and free radicals generates the enormously injurious peroxynitrite, which directly oxidizes low-density lipoprotein and plays a crucial role in the permanent damage of the cell membrane. Therefore, free radicals are scavenged by quercetin and restrained from reacting with NO, whereas, silibin reacts directly with NO (Dehmlow et al., 1996; Shutenko et al., 1999). Mechanical allodynia induced by spinal nerve ligation (SNL) was found to be decreased by various flavonoids including myricetin (Hagenacker et al., 2010), EGCG (Choi et al., 2012), and baicalein (Cherng et al., 2014). SNL-induced thermal hyperalgesia was reduced by myricetin (Hagenacker et al., 2010) and baicalein (Cherng et al., 2014), while quercetin decreased both cold and thermal hyperalgesia in SNL rats (Ji et al., 2017). In addition to this, hesperetin and quercetin decreased partial sciatic nerve ligation-stimulated neuropathic pain and spared nerve injury (Figure 3; Aswar et al., 2014; Muto et al., 2018).

Physiologically, xanthine dehydrogenase plays an important role in the metabolism of xanthine to uric acid, however, this enzyme alters into xanthine oxidase in the case of ischemic-reperfusion, which works as a precursor of free radicals. There are various flavonoids, such as quercetin, silibinin, and luteolin, that are recognized to work as antioxidants through stopping xanthine oxidase (Chang et al., 1993; Shoskes, 1998). Similarly, reperfusion is also caused by the mobilization of leucocytes producing the subsequent release of inflammatory mediators as well as cytotoxic oxidants, which provokes the complement system. Many flavonoids play a key role in the immobilization of leucocytes, eventually resulting in a decline in the serum complement system as well as inflammation (Friesenecker et al., 1995; Ferrándiz et al., 1996). It has been observed that the connection of the same pathophysiological mechanisms takes place with both NP of peripheral origin and inflammation. Both kinds of pathologies express as hyperalgesia and allodynia (Clatworthy et al., 1995; DeLeo and Yezierski, 2001; Jin et al., 2003). Moreover, inflammatory cells infiltration and their main secretory products, including cytokines and arachidonic acid, affect peripheral nerve damage, which is accountable for the production and maintenance of the constant pain (Tracey and Walker, 1995; Cui et al., 2000; Ma and Eisenach, 2003). When cytokines such as IL-1, IL-6, and TNF-α were injected into a rat paw, it would result in the initiation of thermal and mechanical hyperalgesia (Cunha et al., 1992; Ferreira et al., 1993). On the other hand, the inhibition of TNF-α in the animal models with painful neuropathy led to the reduction of hyperalgesia (Sommer et al., 1998). The release of cytokines also activates COX-2 dependent prostanoid releases. Furthermore, prostaglandins (PGs) also play a pivotal role in triggering inflammation that increases sensitivity to pain (Uddin et al., 2020b). It had been found that intrathecal injection of PGs such as PGE_2_ and PGF_2α_ triggered allodynia in conscious mice (Minami et al., 1992, 1994), while intrathecal administration of PGD_2_ and PGE_2_ led to the initiation of hyperalgesia (Uda et al., 1990). Additionally, synthesis of NO and PG through COX-2 as well as iNOS is increased in the microglia on account of peripheral nerve damage, leading to hypersensitization (Hanisch, 2002). It is evident that flavonoids show anti-inflammatory activity both *in vitro* and *in vivo*. One of the imperative mechanisms of anti-inflammatory action is recognized by inhibiting eicosanoid producing enzymes such as phospholipase A2, lipoxygenases, and COX (Kim et al., 2004). Along with anti-inflammatory activity, flavonoids also block arachidonic acid metabolism (Ferrándiz and Alcaraz, 1991).

## Conclusion

In this review, we discuss the effects of flavonoids in improving different NP conditions and how flavonoids control diverse pain biomarkers in animal models of NP. Allosteric modulators at GABA_A_ receptors can alter either the affinity or efficacy of agonists including GABA, subsequently controlling their activity. Flavonoids are strong allosteric modulators and may serve as valuable candidates in the management of NP. Hence, it can be said that there is huge potentiality in flavonoids for the development of novel therapeutics agents for NP, however, further studies are needed.

## Author Contributions

MU conceived the original idea and designed the outlines of the study. MU, AM, MR, and MK wrote the draft of the manuscript. MU and AM prepared the figures for the manuscript. SA, IA, AP, GA, MB-J, and MA-D revised and improved the draft. All authors have read and approved the final manuscript.

## Conflict of Interest

The authors declare that the research was conducted in the absence of any commercial or financial relationships that could be construed as a potential conflict of interest.

## Abbreviations

BDZ: benzodiazepines
CNS: central nervous system
CIPN: chemotherapy-induced peripheral neuropathy
CCI: chronic constriction injury
EGCG: epigallocatechin gallate
GABA: γ -amino butyric acid
MDA: malondialdehyde
NMDA: N-methyl -D-aspartate
NP: neuropathic pain
NF- κ B: nuclear factor kappa B
Nrf2: nuclear factor erythroid 2-related factor 2
SDH: spinal dorsal horn
SNL: spinal nerve ligation
SSNS: somatosensory nervous system.
